# New impact factor and PubMed Central Service from the *Cardiovascular Journal of Africa*

**Published:** 2012-08

**Authors:** Andries Brink

**Affiliations:** Cardiovascular Journal of Africa

The *Cardiovascular Journal of Africa* is pleased to announce an increase in its 2011 impact factor to 0.767, as provided by Thompson Reuters (ISI). Currently, we rank 161 out of 273 indexed journals in the field of cardiology and cardiovascular medicine throughout the world, according to the Scopus journalrating system. The *Cardiovascular Journal of Africa* is truly entrenched in Africa and worldwide

In order to improve visibility for our authors, the *Cardiovascular Journal of Africa* has developed the capability of publishing your article to PubMed Central (PMC), which is the free electronic archive of the *Biomedical and Life Sciences Journal* literature at the US National Institutes of Health National Library of Medicine (NIH/NLM). Currently your article is available on PubMed as a PDF from Sabinet e-publications. PubMed Central is therefore an additional repository of your full-text article. This ensures a long existence for your research and availability for generations to come.

The further value of PMC lies in its capacity to store and cross reference data from diverse sources using a common format within a single repository. With PMC, a user can quickly search the entire collection of full-text articles and locate all relevant material. PMC also allows for the integration of its literature with a variety of other information resources that can enhance the research and knowledge fields of scientists, clinicians and others. We are offering authors from our 2012 published articles first opportunity to use this service at an initial fee of 80 US dollars.

Our authors will also be pleased to note that PubMed will now index our electronic journal. This allows us to publish more articles as we are not restricted by print dynamics. Since listing in Index Medicus, PubMed in 2007 as the *Cardiovascular Journal of Africa*, 590 peer-reviewed articles have been published and are freely available as linked full text.

Authors and reviewers deserve our gratitude as their efforts help to record the development of cardiovascular research across our continent. The *Cardiovascular Journal of Africa* receives articles from across the world, but gives priority to articles from Africa.

In order to assist Francophone Africa, we are enlisting French reviewers with competency in cardiovascular medicine. We need to expand this list, so if you are able to assist in reviewing, please contact our development editor, Glenda Hardy on Glenda@clinicscardive.com or go online to www.cvja.co.za and register as a reviewer.

The *Cardiovascular Journal of Africa* will be present at the forthcoming PASCAR conference in Dakar, Senegal, 15–20 May 2013. We look forward to seeing you there, and of course, also at the 6th World Congress of Paediatric Cardiology and Cardiac Surgery, 17–22 February 2013 in Cape Town.

